# Characterization and antibacterial efficacy of the broad-host-range phage P108 against biofilm-forming and methicillin-resistant *Staphylococcus aureus*

**DOI:** 10.1016/j.virusres.2026.199745

**Published:** 2026-05-12

**Authors:** Xuemei Wei, Ruiyang Zhang, Jianglin Liao, He Liu, Zhen Hu, Weilong Shang, Jing Wang, Ming Li, Xiancai Rao, Shuguang Lu

**Affiliations:** a Medical Research Institute, Southwest University, Chongqing 400716, China; b Department of Microbiology, College of Basic Medical Sciences, Army Medical University, Key Laboratory of Microbial Engineering under the Educational Committee in Chongqing, Chongqing 400038, China; c Department of Cardiovascular Medicine, Center for Circadian Metabolism and Cardiovascular Disease, Southwest Hospital, Army Medical University, Chongqing 400038, China; d Department of Cardiology, Xinqiao Hospital, Army Medical University, Chongqing 400037, China

**Keywords:** Methicillin-resistant *Staphylococcus aureus* (MRSA), Phage P108, Genomic analysis, Broad host range, Antibacterial efficacy, Antibiofilm activity

## Abstract

*Staphylococcus aureus* (*S. aureus*) is a widely distributed opportunistic pathogen capable of causing a variety of serious infections, with its antibiotic resistance becoming increasingly prevalent. As natural bactericidal agents, bacteriophages (phages) have emerged as promising therapeutic alternatives to antibiotics. In this study, we isolated a lytic phage P108 capable of targeting methicillin-resistant *S. aureus* (MRSA) strains. Phage P108 features an icosahedral head with a diameter of approximately 84.7 nm and a contractile tail measuring about 221.5 nm in length. It has a latent period of 20 min and completes lysis within 60 min. Whole-genome sequencing revealed a linear dsDNA genome of 140,807 bp, encoding 226 putative proteins and 3 tRNAs. Four novel structural protein-coding genes were identified. Phylogenetic analysis demonstrated that P108 represents a member of the *Herelleviridae* family, *Kayvirus* genus. P108 is capable of lysing 79.2% (95/120) of clinical isolates of *S. aureus* and demonstrates broad-spectrum lytic activity against MRSA (84.8%, 39/46). Furthermore, phage P108 exhibits high stability, potent *in vitro* bactericidal activity, and effective bacterial biofilm removal, outperforming vancomycin in overall efficacy. These findings highlight its potential for antibacterial applications and support its development as a novel therapeutic strategy against drug-resistant *S. aureus* infections.

## Highlights

• A lytic myovirus, phage P108, capable of targeting methicillin-resistant *Staphylococcus aureus* (MRSA) strains, was isolated and characterized.
• Phylogenetic analysis revealed that phage P108 represents a novel member of the *Kayvirus* genus within the *Herelleviridae* family.
• Phage P108 effectively lyses 79.2% (95/120) of clinical *S. aureus* isolates, with a notably high lysis rate of 84.8% (39/46) against MRSA.
• Phage P108 exhibits high stability, potent *in vitro* bactericidal activity, and effective bacterial biofilm clearance, outperforming vancomycin in overall efficacy.

## 1 Introduction

*Staphylococcus aureus* (*S. aureus*) is an important opportunistic pathogen commonly found on the skin surface and nasopharyngeal mucosa, and it is closely associated with both nosocomial and community-acquired infections (Alexander et al., 2023; Melocco et al., 2025). This bacterium can cause a wide range of infectious diseases, including common acute skin and soft tissue infections, as well as severe conditions such as necrotizing pneumonia, endocarditis, sepsis, and osteomyelitis, posing a significant threat to human health (Fu et al., 2025; Sharma et al., 2022). With the widespread and often inappropriate use of antibiotics, the issue of drug resistance in *S. aureus* has become increasingly prominent. The first methicillin-resistant *S. aureus* (MRSA) strain was identified and reported in 1961, just two years after the clinical introduction of methicillin in 1959. Since then, MRSA has rapidly spread worldwide and has emerged as one of the major global public health challenges of the 21st century (Lakhundi and Zhang, 2018; Miller and Arias, 2024). According to the Lancet report, drug-resistant infections were responsible for 4.95 million deaths globally in 2019, with drug-resistant *S. aureus* infections ranking as the second deadliest cause (Collaborators, 2022). Currently, vancomycin serves as the “last line of defense” in the clinical treatment of *S. aureus* infections. However, the successive emergence and spread of vancomycin-intermediate *S. aureus* (VISA) and vancomycin-resistant *S. aureus* (VRSA) have posed new challenges to clinical management (Gardete and Tomasz, 2014). As early as 2017, the World Health Organization listed MRSA as one of the key drug-resistant pathogens in urgent need of exploration and development of new antibacterial agents (Jubeh et al., 2020). In addition, *S. aureus* is prone to form biofilms, which can block the penetration of antibiotics and escape the immune clearance of the host (Idrees et al., 2021; Olsen, 2015). Therefore, there is a huge practical demand for the development of new anti-*S. aureus* drugs, and there is an urgent need to find new strategies for the control of drug-resistant *S. aureus* infections.

Bacteriophages (phages), often referred to as the “natural killers” of bacteria, have been utilized to treat bacterial infections since their discovery (Macnair et al., 2024; Weiner et al., 2025). Phages exhibit strict host specificity, selectively targeting and lysing pathogenic bacteria without disrupting the host’s normal microbiota, making them a promising alternative therapeutic agent for MRSA infections (Düzgünes et al., 2021). The development of new antibiotics is both costly and time-consuming, whereas phage resources are abundant. For instance, seawater alone contains approximately 10^7^ phage particles per milliliter, with an estimated global phage population of 10^31^, providing an extensive reservoir of potential antibacterial agents (Hatfull et al., 2022). Recent studies have demonstrated that phages exhibit significant therapeutic efficacy against *S. aureus*-induced skin, lung, and chest infections in various animal models and human clinical trials (Liu et al., 2023a; Petrovic Fabijan et al., 2020).

However, *S. aureus* phage therapy still faces several challenges and limitations. On the one hand, the existing *S. aureus* phage library is relatively limited, and some phages exhibit issues in clinical application, such as a narrow host range, unstable lysis efficiency, reduced efficacy against biofilm-associated bacteria, and potential bacterial resistance (Djebara et al., 2025; Petrovic Fabijan et al., 2020). On the other hand, the methods for isolating and identifying *S. aureus* phages require optimization, and many aspects of their biological characteristics, genomic information, and interaction mechanisms with host bacteria remain poorly understood, which limits the further development and broader application of *S. aureus* phage therapy (Strathdee et al., 2023). Therefore, it is crucial to isolate and identify new *S. aureus* phages (Hegarty, 2025). The aim of this study was to isolate, identify, and characterize a new phage of *S. aureus* and evaluate its antimicrobial potential. Through morphological observation, host range analysis, whole-genome sequencing, structural protein studies, *in vitro* antibacterial and anti-biofilm activity assays, the biological and genomic characteristics and antibacterial application potential of phage P108 were elucidated, so as to provide new resource for *S. aureus* phage therapy and insights into the development of novel antibacterial agents.

## 2 Materials and methods

### 2.1 Bacterial strains and sample sources

The MRSA strain XN108 was isolated from clinical samples at Southwest Hospital in Chongqing, China (Rao et al., 2022; Zhang et al., 2013), with a vancomycin (VAN) minimum inhibitory concentration (MIC) of 12 µg/mL, classifying it as a vancomycin-intermediate *Staphylococcus aureus* (VISA) strain. Additional 119 *S. aureus* strains were collected from multiple hospitals across Chongqing, Tianjin, and Guangzhou (Liu et al., 2024a, 2023b; Rao et al., 2022). The phage P108 was isolated from sewage samples obtained from Southwest Hospital in Chongqing.

### 2.2 The cultivation of bacteria

The bacteria were cultured as described previously (Liu et al., 2024a, 2023b). Briefly, the stored XN108 strain was retrieved from liquid nitrogen and inoculated onto a solid brain heart infusion (BHI, Oxoid, UK) agar plate. The plate was incubated overnight at 37 °C to obtain single colonies. The following day, a single colony of *S. aureus* was selected using a sterile inoculation loop and transferred into 3 mL of liquid BHI medium. The culture was incubated under shaking conditions (37 °C, 220 rpm) for approximately 5 h until it reached a bacterial concentration of approximately 1 × 10^8^ colony forming units (CFU)/mL, then stored at 4 °C as the bacterial stock solution. For subsequent experiments, 10 μL of the stock culture was inoculated into 3 mL of fresh liquid BHI medium and incubated (37 °C, 220 rpm) until the OD_600_ reached approximately 0.3 (for phage infection assays) or 0.5 (for double-layer agar plaque assays).

### 2.3 Phages isolation

The separation of phages was carried out by referring to the method described previously (Lu et al., 2013), with slight changes. 200 μL of XN108 culture was added into 200 mL BHI liquid medium. The culture was shaken at 37 °C and 220 rpm for approximately 5 h until the OD_600_ reached approximately 0.5. Subsequently, 200 mL of sewage was added to the above mixture, and after incubation for an additional 5 h, 200 mL of BHI liquid medium was supplemented, followed by overnight cultivation at 37 °C and 220 rpm. Finally, 2 mL of the cultured sewage mixture was collected and centrifuged at 5000 g for 10 min. Then, the supernatant was filtered through a 0.22 μm sterile needle filter, and the filtrate was stored for standby. Five centrifuge tubes (15 mL) were prepared, each inoculated with 200 μL of the XN108 bacterial culture. Subsequently, 200 μL and 10 μL of filtrate were added to designated tubes, along with 10 μL of 10^−1^ and 10^−2^ diluted filtrate, respectively. 10 μL of BHI medium was set as control. The mixtures were vortexed thoroughly and incubated at room temperature for 15 min. Approximately 3 mL of melted BHI semi-solid medium was then added to each tube, immediately vortexed to ensure uniform mixing, and promptly poured onto BHI solid agar plates. After the overlay solidified, the plates were inverted and incubated at 37 °C overnight. The formation of plaques was examined the following day.

### 2.4 Purification of phage particles

The phage was purified following a previously reported method (Tang et al., 2018). A single colony of the host bacterium XN108 was inoculated into 300 mL of liquid BHI medium and incubated at 37 °C with shaking at 220 rpm for approximately 3∼4 h. A single plaque of phage P108 was picked and added to the bacterial culture, which was then incubated at 37 °C and 220 rpm for 5 h, followed by overnight incubation at room temperature until the lysate became clear. DNase I (5 μg/μL) and RNase A (10 μg/μL) were added to the culture to achieve a final concentration of 1 μg/mL each, and the mixture was incubated in a water bath at 37 °C for 30 min. NaCl was added to a final concentration of 5.84 g/100 mL, mixed thoroughly until dissolved, and the solution was placed on ice for 1 h. The sample was then centrifuged at 10,000 × g for 10 min, and the supernatant was collected. Solid polyethylene glycol (PEG) 8000 was added to the supernatant to reach a final concentration of 10% (w/v). After vigorous shaking to ensure complete dissolution, the solution was incubated on ice for 1 h to facilitate phage particle precipitation. This was followed by centrifugation at 12,000 × g and 4 °C for 10 min. The pellet was resuspended in approximately 2.0 mL of TM (tris-magnesium sulfate) buffer (pH 7.5). An equal volume of chloroform was added, gently vortexed for 30 s, centrifuged at 5000 × g for 10 min, and the upper aqueous phase was collected. This constituted the crude phage P108 preparation.

### 2.5 Transmission electron microscopy (TEM) imaging

Transmission electron microscopy observation of phage particles was performed as previously described (Lu et al., 2013). A 20 µL drop of purified phage P108 suspension (∼10^10^ plaque-forming units (PFU)/mL) was placed on a paraffin sheet. A 200-mesh carbon-coated copper grid was then immersed in the droplet and allowed to adsorb for approximately 15 min. The grid was carefully removed and air-dried for 2–3 min. Subsequently, it was stained with 2% (m/v) sodium phosphotungstate solution (pH 7.6) for 2 min. Finally, the morphology of the phage was examined using transmission electron microscopy (Hitachi H600A, Japan) at an accelerating voltage of 80 kV and a magnification of 200,000 × .

### 2.6 Determination of the stability of phage P108

The stability of phage P108 was evaluated according to previously reported methods (Peters et al., 2023; Wang et al., 2024). For pH stability, 1 mL of phage P108 suspension (∼10^9^ PFU/mL) was transferred into 12 separate microcentrifuge tubes, and 1 mL of PBS buffer at pH values of 1, 2, 3, 4, 5, 6, 7, 8, 9, 10, 11, and 12 was added to each tube, respectively. The mixtures were incubated at 37 °C for 1 h, after which the phage titers were determined using a dot assay. For thermal stability, 1 mL of phage P108 suspension (∼10^9^ PFU/mL) was placed in individual test tubes and incubated in a constant-temperature metal bath at 4 °C, 37 °C, 50 °C, 60 °C, and 70 °C for 1 h. After treatment, phage titers were assessed by dot assay. To evaluate chloroform resistance, 500 μL of filtered phage P108 suspension (∼10^9^ PFU/mL) was mixed with 500 μL of chloroform in a sterile EP tube, gently vortexed for 30 s, and centrifuged at 5000 × g for 1 min. The upper aqueous phase was carefully collected as the chloroform-treated phage sample, and its titer was determined by dot assay. For UV irradiation sensitivity, 1 mL aliquots of phage P108 suspension (∼10^9^ PFU/mL) were dispensed into 2 mL centrifuge tubes and exposed to UV irradiation for 10, 20, 30, 40, 50, and 60 min, respectively. The phage titers were then quantified using the double-layer agar plate method (Wang et al., 2024). All experiments were performed in triplicate.

### 2.7 Host spectrum determination

The host spectrum of phage P108 was determined according to a previously described method (Liu et al., 2024a). Briefly, the standard dot assay was employed for host range analysis. The *Staphylococcus* strains used in this study and their characteristics were as previously reported (Liu et al., 2024a, 2023b; Rao et al., 2022; Zhang et al., 2013). Each test strain was cultured overnight in BHI broth. A 30 μL aliquot of the overnight culture was inoculated into 3 mL of fresh BHI medium and incubated at 37 °C with shaking at 200 rpm for approximately 3.5 h until reaching the logarithmic growth phase (OD_600_ ≈ 0.15). Then, 30 μL of the logarithmic-phase bacterial culture and 30 μL of phage suspension (titer approximately 10^9^ PFU/mL) were mixed, and 10 μL of the mixture was spotted onto a BHI agar plate. After overnight incubation at 37 °C in an inverted position, bacterial growth was observed, and phage-mediated lysis was assessed based on the presence or absence of clearing zones.

### 2.8 Optimal multiplicity of infection (MOI) determination

The concentration of the host bacterial suspension was adjusted to 1 × 10^6^ colony forming units (CFU)/mL in BHI medium. The phage and host bacteria were mixed at different MOI ratios (0.0001, 0.001, 0.01, 0.1, 1, 10, and 100), followed by incubation at 37 °C for 3 min to allow adsorption. After adsorption, the mixture was cultured at 37 °C for 5 h, during which the bacterial culture became visibly clear, indicating lysis. The sample was then centrifuged at 5000 × g for 10 min, and the supernatant was filtered through a 0.22 μm filter. The phage titer in the supernatant was determined using dot assay (Le et al., 2024). The MOI yielding the highest phage titer was defined as the optimal MOI. All experiments were performed in triplicate.

### 2.9 One-step growth curve analysis

The one-step growth characteristics of phages were identified according to the previously reported method (Li et al., 2024b). The phage P108 and host bacteria XN108 were mixed according to the best MOI. After standing at 37 °C for 5 min, the mixture was centrifuged at 5000 g for 10 min. After discarding the supernatant, 5 mL BHI medium was added to suspend and precipitate, and then cultured in 37 °C incubator. Aliquots of 100 µL were collected from the mixture at different time points (0, 10, 20, 30, 40, 50, 60, 70, 80, 90, 100, 110, and 120 min), mixed with 100 µL of host bacteria after serial dilution, and used to prepare double-layer agar plates (Lu et al., 2013; Tang et al., 2018). Each group of experiments was repeated three times in parallel.

### 2.10 Extraction of phage nucleic acid

The nucleic acid of phage P108 was extracted and purified according to a previously reported method (Lu et al., 2013). Purified phage particles were treated with DNase I and RNase A at final concentrations of 5 μg/mL and 1 μg/mL, respectively, and incubated at 37 °C for 1 h. EDTA (ethylene diamine tetraacetic acid) (pH 8.0) was then added to a final concentration of 20 mM to inactivate DNase I. Subsequently, proteinase K was added to a final concentration of 50 μg/mL and sodium dodecyl sulfate (SDS) to a final concentration of 0.5% (w/v), and the mixture was incubated at 56 °C for 1 h. The sample was extracted once with an equal volume of balanced phenol (pH 8.0), centrifuged at 5000 × g for 10 min, and the upper aqueous phase was collected. This was followed by a second extraction with an equal volume of chloroform, centrifugation at 5000 × g for 10 min, and collection of the upper aqueous phase. Nucleic acid was precipitated by adding two volumes of cold anhydrous ethanol, incubating at −20 °C for 1 h, and centrifuging at 12,000 × g for 10 min at 4 °C. The supernatant was carefully removed, and the pellet was air-dried at room temperature. The purified genomic nucleic acid was resuspended in an appropriate volume of TE (tris EDTA) buffer (1.0∼2.0 mL). After quantification using a nucleic acid spectrophotometer, the sample was stored at −20 °C.

### 2.11 Genome sequencing and analysis

Personal Biotechnology Co., Ltd. (Shanghai, China) was commissioned to perform genome sequencing of phage P108. The phage genome sequence was determined by IonTorrent (Thermo Fisher, USA), and the whole genome was assembled by Newbler v.2.8 software. RAST Server (https://rast.nmpdr.org/rast.cgi) (McNair et al., 2018) was employed for P108 genome annotation. Then FGENESV (http://www.softberry.com/berry.phtml?topic=virus&group=programs&subgroup=gfindv), GeneMark (https://exon.gatech .edu/) (Brůna et al., 2024) and BLAST (Singh and Raghava, 2016) were used to verify the annotation results. tRNAscan - SE 2.0 (http://trna.ucsc.edu/tRNAscan-SE/) (Chan et al., 2021; Chan and Lowe, 2019) was used to predict tRNA encoding genes. The annotation information of phage P108 genome was submitted to GenBank (https://www.ncbi.nlm.nih.gov/genbank/) (Sayers et al., 2024) through BankIt (https://www.ncbi.nlm.nih.gov/WebSub/). The genome map of P108 was presented using BRIG (Alikhan et al., 2011) and Proksee (Grant et al., 2023). DeePhage (Wu et al., 2021) (http://cqb2.pku.edu.cn/ZhuLab/DeePhage/) was used to predict phage P108 life cycle. ResFinder 4.6.0 (Bortolaia et al., 2020) (http://genepi.food.dtu.dk/resfinder) was used to predict drug-resistant genes in the genomes of P108. Potential virulence factors encoded in the P108 genome were predicted using VFDB (Liu et al., 2022) (https://www.mgc.ac.cn/cgi-bin/VFs/v5/main.cgi).

### 2.12 Identification of the P108 structural proteins

Identification of phage structural proteins was performed according to previously described methods (Lu et al., 2013). Briefly, purified P108 particles were thermally denatured and subsequently loaded onto a 10% (w/v) polyacrylamide gel for the analysis of P108 structural proteins. After staining with Coomassie Brilliant Blue R250, individual protein bands were excised from the gel and analyzed by high-performance liquid chromatography-mass spectrometry (HPLC-MS) using an HPLC—Chip-MS/MS Ion Trap 6330 system (Agilent, Santa Clara, CA, USA). The resulting data were processed using Agilent Spectrum Mill proteomics software (Rev A.03.02.060; Agilent, Santa Clara, CA, USA) for further analysis.

### 2.13 Multiple sequence alignment and phylogenetic analysis

Based on whole-genome BLAST (Singh and Raghava, 2016) comparisons, 13 phage genomes most similar to P108 were selected. These phage genomes were analyzed using EasyFig (Sullivan et al., 2011) (http://mjsull.github.io/Easyfig/) for tBLASTX comparisons and generation of linear comparison maps. BRIG (BLAST Ring Image Generator, https://sourceforge.net/projects/brig/) (Alikhan et al., 2011) comparison of complete amino acid sequences of JP4 with other 13 related phages was performed. Additionally, the VICTOR online tool (Meier-Kolthoff and Göker, 2017) (https://ggdc.dsmz.de/victor.php) were used to analyze 52 phage genomes that had BLAST (Singh and Raghava, 2016) maximum scores exceeding 35,000 relative to P108, and a phylogenetic tree was constructed based on these analyses. The proteomic phylogenetic tree of phage P108 and other dsDNA phages was constructed using the ViPTree server (version 4.0) (Nishimura et al., 2017a, 2017b). A total of 5633 dsDNA phage genomes (Table S1), including that of P108, were included in the analysis. The analysis was performed using the default parameters provided by the server. Taxonomic classification of phage P108 and the calculation of average nucleotide identity (ANI) were performed using taxMyPhage (https://ptax.ku.dk/).

### 2.14 *In vitro* antibacterial activity assessment and biofilm eradication assay

The experimental procedures were adapted from previously reported methods with minor modifications (Li et al., 2024b; Lu et al., 2021). The host strain XN108 was cultured to the logarithmic growth phase (∼10^5^ CFU/mL). Phage P108 and bacteria were mixed at MOIs of 0.1, 1, and 10, respectively. PBS buffer was used as the negative control. For the positive control group, vancomycin (VAN) was added to the XN108 bacterial suspension at final concentrations of 12 μg/mL (1 × MIC), 24 μg/mL (2 × MIC), and 48 μg/mL (4 × MIC). After thorough mixing, the samples were incubated at 37 °C, and the OD_600_ value was measured hourly over a 12 h period. The biofilm-clearing effect of phage P108 on XN108 was evaluated using crystal violet staining method (Li et al., 2024b; Lu et al., 2021). Briefly, *S. aureus* XN108 was cultured to the logarithmic phase (approximately 10^5^ CFU/mL). A volume of 100 μL of the bacterial suspension was added to 100 μL of fresh BHI medium in a 96-well plate and incubated at 37 °C for 24 h to allow biofilm formation. After that, the medium was removed, and the wells were washed twice with PBS and air-dried at room temperature. Subsequently, 200 μL of phage P108 (10^5^∼10^9^ PFU/mL) or vancomycin (1 × MIC, 2 × MIC, and 4 × MIC) was added to each well. After incubation at 37 °C for 1 h, the supernatant was discarded, and the wells were washed twice with PBS and air-dried. Each well was then stained with 200 μL of 0.1% crystal violet solution for 15 min. The unbound dye was removed by discarding the supernatant, followed by two PBS washes and natural air-drying. To solubilize the bound dye, 200 μL of 33% acetic acid was added to each well and incubated for 30 min with gentle shaking. The absorbance at 570 nm (OD_570_) was measured using a microplate reader (Bio-Rad, USA). The PBS-treated group served as the negative control. All experiments were performed in triplicate.

### 2.15 Statistical analysis

Experimental data are presented as mean ± standard deviation (SD), with n = 3. Each experiment was performed in triplicate, and the average values were used for analysis. Data were analyzed using one-way ANOVA with GraphPad Prism 9.0 software. Error bars represent the standard deviation (SD). A *p*-value of <0.05 was considered statistically significant, while *p* < 0.01 or lower indicates a highly significant difference.

## 3 Results

### 3.1 Phage isolation and morphology

**Fig. 1:**
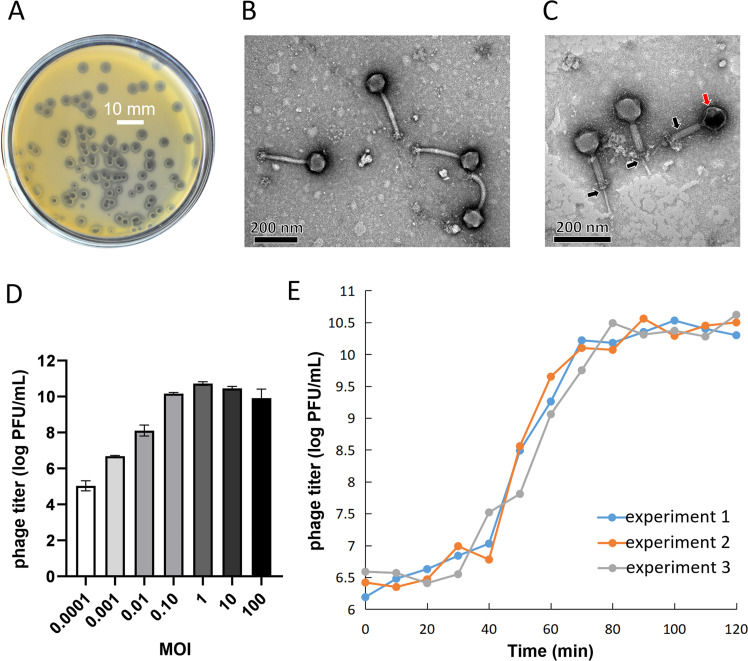
Biological characteristics of phage P108. **(A)** Plaque morphology of P108. Scale bar: 10 mm. **(B)** TEM morphology of intact P108 particles. Scale bar: 200 nm. **(C)** TEM morphology of contracted P108 particles. The black arrow points to the contracted tail, whereas the red arrow indicates the emptied head. Scale bar: 200 nm. **(D)** MOI determination of phage P108. **(E)** One-step growth curve of phage P108. The experiment was repeated in triplicate.

On the bacterial lawn, phage P108 forms transparent plaques with a diameter of approximately 4 mm , each surrounded by a semi-transparent halo (Fig. 1A). Transmission electron microscopy revealed that P108 particles have an icosahedral head measuring approximately 84.7 nm in diameter and a contractile tail measuring about 221.5 nm in length (Fig. 1B and C). These characteristics indicated that P108 is a lytic phage that infects *S. aureus*.

### 3.2 Optimal MOI and growth characteristics

To determine the optimal multiplicity of infection (MOI), host bacterium XN108 was infected with phage P108 at various MOIs, and the phage titer was measured after 5 h of incubation. The results showed that the phage P108 titer peaked at 5.5 × 10^10^ PFU/mL (Fig. 1D) when the MOI was 1, indicating that an MOI of 1 is optimal for phage P108. Subsequently, phage P108 and host bacterium XN108 were co-cultured at an MOI of 1, and the phage titer was monitored at regular intervals to construct a one-step growth curve for P108 (Fig. 1E). The data revealed a latent period of approximately 20 min, followed by lysis of the host bacterium and release of P108 progeny within 60 min (Fig. 1E). Lysis of XN108 cells infected with phage P108 resulted in an average burst size of 83 viral particles per cell.

### 3.3 P108 phage host range

**Fig. 2:**
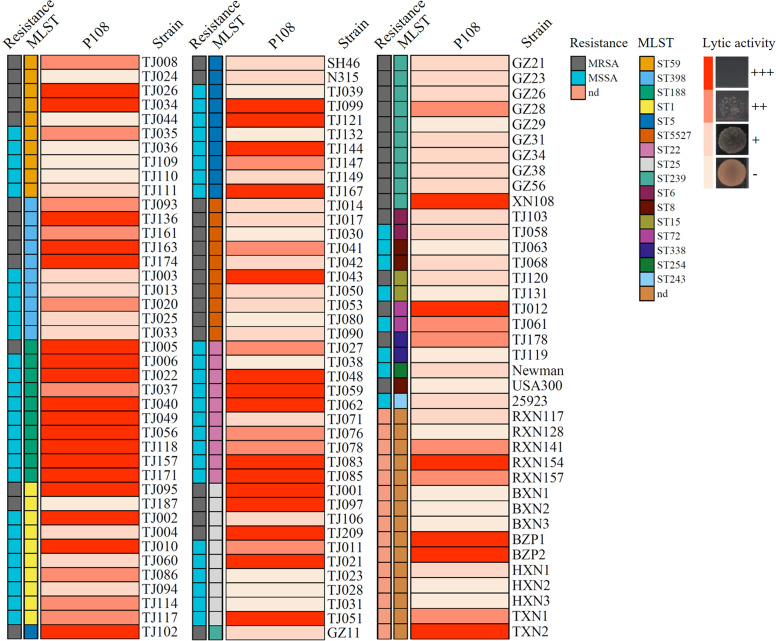
Host spectrum of phage P108. MLST: multilocus sequence typing. MRSA: methicillin-resistant *Staphylococcus aureus*. MSSA: methicillin-sensitive *Staphylococcus aureus*. nd: not determined. +++: reduction of >4 log10 units. ++: reduction of 2–4 log10 units. +: reduction of 0.5–2 logl0 units. -: Reduction of <0.5 log_l0_ units.

A dot assay was used to evaluate the lytic activity of phage P108 against 120 clinical strains of *S. aureus* belonging to different sequence types (ST). The results showed that phage P108 could lyse 95 out of the 120 strains (“+” and above, 79.2%) (Fig. 2). Among these strains, 39 exhibited a reduction of approximately four orders of magnitude in bacterial counts following exposure to phage P108, leading to complete bacterial lysis (+++) (Fig. 2). Among all the tested strains, there were 46 MRSA strains (Fig. 2). Of the 46 MRSA strains, 39 were lysed (“+” and above, 84.8%), with 14 showing complete lysis (“+++”, 30.4%) (Fig. 2), indicating broad-spectrum lytic activity against MRSA.

### 3.4 Physicochemical stability of P108 phage under experimental conditions

**Fig. 3:**
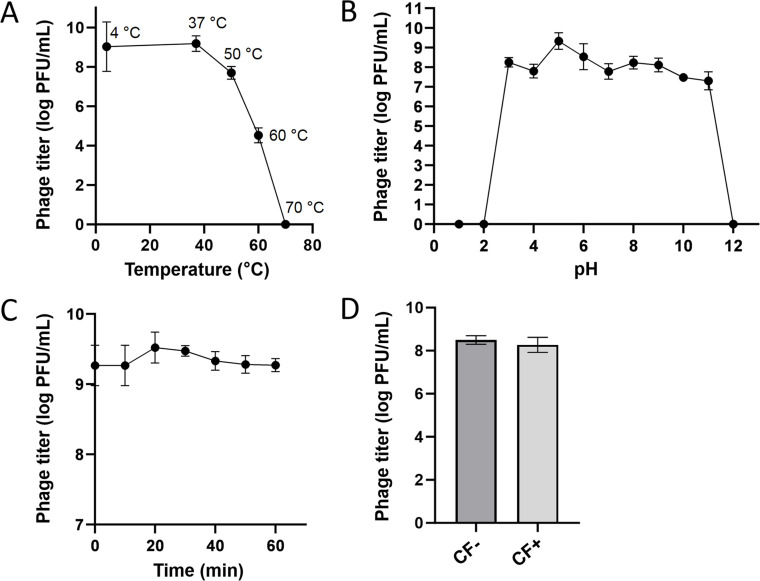
The stabilityof phage P108. **(A)** Thermal stability of P108. **(B)** pH stability of P108. **(C)** Ultraviolet (UV) sensitivity of P108. The horizontal axis denotes the duration of UV irradiation. **(D)** Chloroform sensitivity of P108. CF: chloroform.

The titers of phage P108 remained almost unchanged when incubated at 4 °C and 37 °C for 1 h, with titers of approximately 10^8^ PFU/mL in both cases. Increasing the temperature to 50 °C resulted in only a slight decrease in P108 activity, while at 60 °C, the titer was reduced to about 10^4^ PFU/mL (Fig. 3A). Phage P108 exhibited stable activity across a pH range of 3 to 11 (Fig. 3B). Exposure to ultraviolet (UV) light caused a gradual decrease in P108 titer over time; however, the titer remained at about 10^9^ PFU/mL after 60 min of exposure (Fig. 3C). Effective plaque formation of phage P108 was observed both with and without chloroform treatment (Fig. 3D), indicating that P108 is not sensitive to chloroform and lacks a lipid outer membrane structure.

### 3.5 The genome of phage P108

**Fig. 4:**
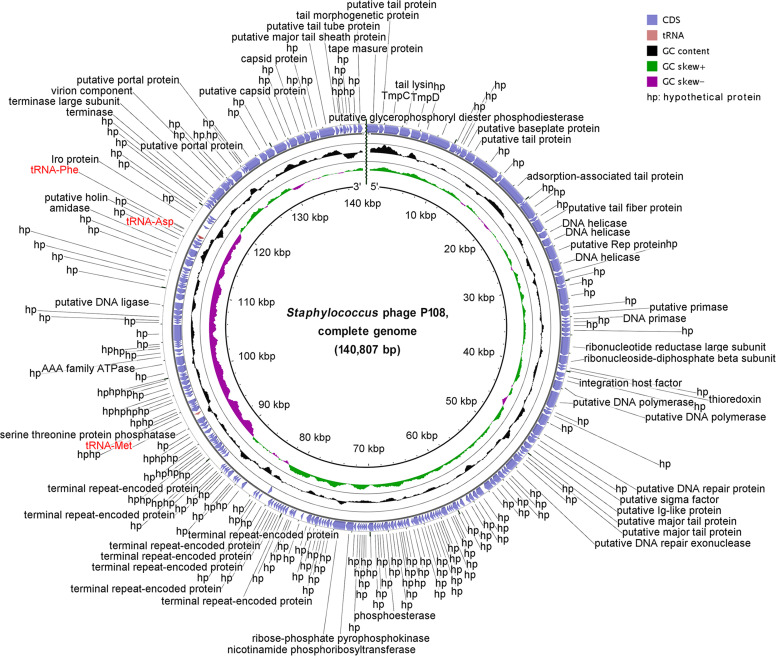
The genomic map of phage P108. The outermost circle represents genes encoded by the positive strand, followed by those encoded by the negative strand.

Whole-genome sequencing and analysis of phage P108 revealed that its genome is a linear double-stranded DNA (dsDNA) molecule with a total length of 140,807 bp (Fig. 4). The genome has a G + C content of 30.22% and encodes 226 proteins and 3 tRNAs. Among the encoded proteins, 100 proteins (44.2%) had predicted functions based on homology alignment. Notably, no integrase, excisionase, transposase, or other related genes were identified in the P108 genome, and no genes associated with drug resistance or virulence factors were detected.

### 3.6 Structural protein analysis of phage P108

**Fig. 5:**
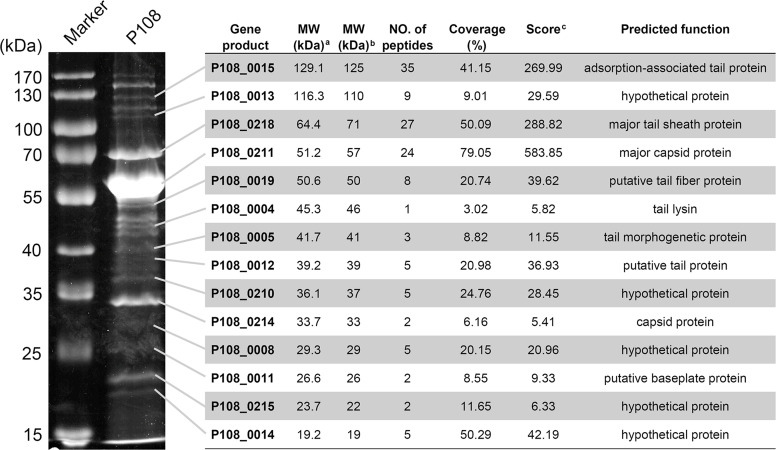
Identification of P108 structural proteins. ^a^MW value was theoretically calculated. ^b^MW value was experimentally estimated. ^c^Distinct summed MS/MS search score.

Sodium dodecyl sulfate-polyacrylamide gel electrophoresis (SDS-PAGE) was used to visualize the structural proteins of phage P108, revealing approximately 20 protein bands with molecular weights ranging from 19 kDa to 170 kDa (Fig. 5). These protein bands were excised for high-performance liquid chromatography-mass spectrometry (HPLC-MS) analysis, and a total of 14 protein bands were identified as coding products of different genes (Fig. 5). The predominant protein band corresponded to the major capsid protein (P108_0211, 57 kDa), consistent with predictions. Additionally, five hypothetical proteins were isolated by SDS-PAGE: P108_0008, P108_0013, P108_0014, P108_0210, and P108_0215, confirming their role as structural proteins (Fig. 5).

### 3.7 Comparative genomic analysis of phage P108

**Fig. 6:**
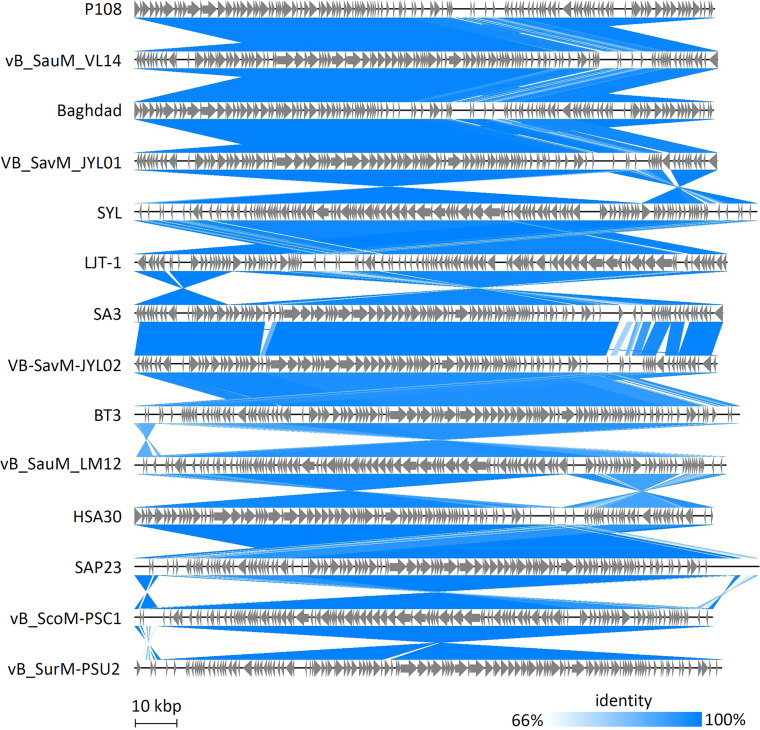
Visual comparison of phage genomes. All the phages shown are *Staphylococcus aureus* phages. The identity cut-off is set at 66%.

**Fig. 7:**
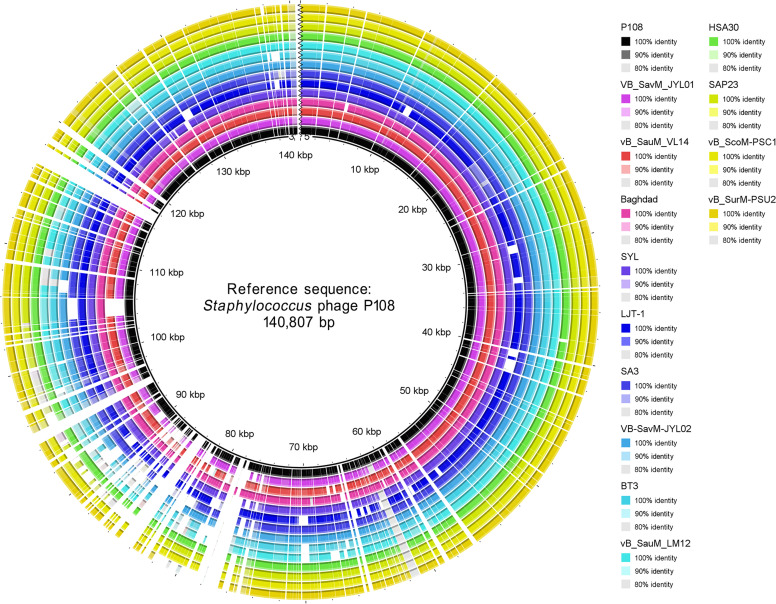
BRIG comparison of complete amino acid sequences of P108 with other 13 related phages showed in Fig. 6. Identity labels of the 14 phage proteomes are shown in the same order as the rings from the innermost to the outermost.

BLASTN analysis of the genome sequence of phage P108 revealed that it is a new member of the family *Herelleviridae* and the genus *Kayvirus*. Thirteen *S. aureus* phages with high sequence similarity to P108 were selected for alignment analysis. The results showed that approximately 15% of their genome regions exhibited mutations, while other regions remained relatively conserved (Fig. 6). Additionally, these genome sequences displayed shifts, inversions, and reverse complements (Fig. 6). To better visualize the variations among these phages, a BRIG alignment based on the complete amino acid sequences was performed (Fig. 7). The results revealed that although these phages share notable sequence similarities, they also exhibit distinct differences (represented as blank regions) that may reflect evolutionary recombination and selective pressures.

### 3.8 Phylogenetic and taxonomic analysis of phage P108

**Fig. 8:**
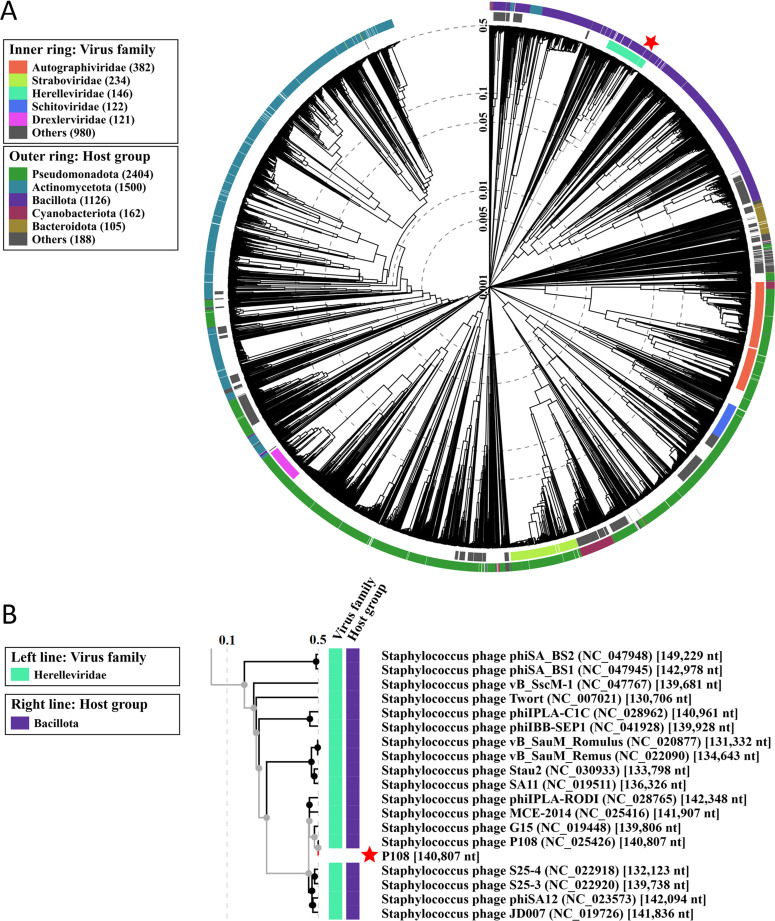
Proteomic phylogenetic tree of phage P108. **(A)** The circular viral proteomic tree of P108. **(B)** A portion of the rectangular proteomic tree illustrating the phages most closely related to P108.

Phylogenetic analysis based on the whole-genome sequence indicated that P108 is most closely related to *S. aureus* phage Baghdad and exhibits a certain genetic distance from other phages (Fig. S1). Proteomic phylogenetic analysis of phage P108 revealed that it clusters within the *Herelleviridae* family (Fig. 8A). This clade comprises 146 members, all of which locate at a monophyletic group in the phylogenetic tree. A closer examination of the proteomic phylogenetic tree indicates that phage P108 is most closely related to *Staphylococcus* phage G15 (Fig. 8B), a topology that differs from the evolutionary tree inferred from whole-genome sequences (Fig. S1). Taxonomic analysis using taxMyPhage revealed that phage P108 shares average nucleotide identity (ANI) values ranging from 80% to 99% with members of the *Kayvirus* genus (Fig. S2), satisfying the International Committee on Taxonomy of Viruses (ICTV) criteria for genus-level classification. The combined genomic, phylogenetic, and taxonomic evidence supports the classification of phage P108 as a member within the *Kayvirus* genus.

### 3.9 *In vitro* antibacterial and antibiofilm activity of P108 phage

**Fig. 9:**
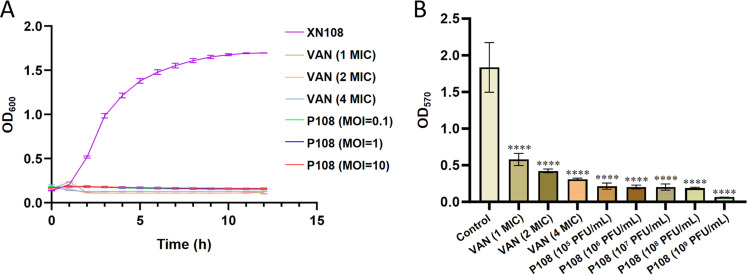
The *in vitro* antibacterial and anti-biofilm activity of phage P108. **(A)** The time-kill curve of phage P108. VAN: vancomycin; MIC: minimum inhibitory concentration. **(B)** The biofilm removal test. ****: *P* < 0.0001.

Under the treatment of phage P108, MOI of 0.1, 1 and 10 could effectively inhibit the growth of host bacterium XN108. When treated with vancomycin of different concentrations (1 MIC, 2 MIC and 4 MIC), the growth of bacteria XN108 was also effectively inhibited. The bacteriostatic effect of phage treatment group and vancomycin treatment group was the same, and the bacterial concentration was much lower than that of the control group (Fig. 9A). Moreover, within 12 h of the test, the growth of drug-resistant bacteria and phage resistant bacteria was not detected in the phage treated group as in the vancomycin treated group. Vancomycin treatment with 1 MIC, 2 MIC and 4 MIC could significantly inhibit the biofilm of MRSA XN108 (>65% biofilm could be removed), and phage P108 of 10^5^–10^9^ PFU/mL could also significantly inhibit the biofilm of XN108 (>80% biofilm could be removed) (Fig. 9B). In general, phage P108 has a better inhibitory effect on biofilm than vancomycin (Fig. 9B).

## 4 Discussion

Studies have shown that MRSA is resistant to nearly all β-lactam antibiotics, including penicillins, cephalosporins, and carbapenems (Hassoun et al., 2017). Glycopeptide antibiotics such as vancomycin serve as the “last line of defense” for treating MRSA and other drug-resistant bacterial infections in clinical settings. However, with the increasing use of vancomycin, vancomycin-intermediate *S. aureus* (VISA) and vancomycin-resistant *S. aureus* (VRSA) have emerged successively (Cong et al., 2020). The host bacterium of phage P108, *S. aureus* XN108, is not only a MRSA strain but also a VISA strain (Rao et al., 2022). Infections caused by this strain pose a significant challenge due to their resistance to common antibiotics, necessitating the development of new antibacterial strategies. However, developing a new antibiotic is time-consuming, economically costly, and often results in mismatched investment and returns (Jubeh et al., 2020). Consequently, phage therapy has garnered renewed attention as a promising alternative to traditional antibiotics (Miller and Arias, 2024; Subedi and Barr, 2021). Multiple preclinical studies in animal models and a limited number of clinical trials have demonstrated the therapeutic efficacy of selected phages against *S. aureus* (including MRSA) infections (Parsons et al., 2026). Nevertheless, the narrow host range of phages and the capacity of *S. aureus* to rapidly evolve resistance underscore the need for robust, large-scale clinical data to establish phage therapy as a reliable and scalable treatment modality for these infections (Strathdee et al., 2023).

The isolation and identification of phages form the foundation and prerequisite for phage therapy. Isolating phages with strong lytic activity, broad host range, good stability, safety, and efficacy provides a critical guarantee for successful phage therapy (Leitner and Mccallin, 2024) and offers a suitable chassis for phage engineering (Yehl et al., 2019). Phage P108 produces large, transparent plaques on bacterial lawns, and no genes related to lysogenic conversion were identified in its genome, indicating that it is a strictly lytic phage without a lysogenic life cycle. For phage therapy, lytic phages are generally preferred (Liu et al., 2024b). In cases where lysogenic phages are isolated, they often need to be converted into lytic phages for clinical application (Cristinziano et al., 2024; Dedrick et al., 2019). Additionally, phage P108 can lyse host bacteria and release progeny phages within 60 min, achieving a progeny phage titer exceeding 10^10^ PFU/mL. Moreover, P108 exhibits good physical and chemical stability, and no genes associated with drug resistance or virulence factors were detected in its genome, supporting its potential as a safe and effective candidate for phage therapy.

The host profile of a phage refers to the species of bacteria that the phage can effectively infect and is a key factor in determining its therapeutic potential. Phages exhibit strict host specificity, typically infecting only one or a few isolates of the same bacterial species. This specificity is mainly determined by the receptor-binding proteins on the phage surface and bacterial defense mechanisms, with only a few phages capable of infecting multiple strains within the same genus or even different genera (Strathdee et al., 2023). Due to the significant variability among clinical isolates within the same species, a phage effective in one patient may not be effective in another. Consequently, host specificity has become a major obstacle to the application of phages in combating drug-resistant bacterial infections (Hatfull et al., 2022; Liu et al., 2023a). Screening for phages with broad host ranges is an effective strategy to address this limitation. To evaluate the host range of phage P108, 120 clinical isolates of *S. aureus* collected from hospitals in Chongqing, Tianjin, and Guangzhou were tested. The results demonstrated that phage P108 exhibited a broad host spectrum, effectively lysing 95 out of 120 bacterial isolates (79.2%), with a particularly high lysis rate of 84.8% (39/46) observed against methicillin-resistant *S. aureus* (MRSA). This host range was comparable to that of *S. aureus* phage Stau2 (164/205, 80%) (Hsieh et al., 2011) and nearly twice as broad as that of *S. aureus* phage JD419 (61/138, 44.2%) (Feng et al., 2021).

*S. aureus* phages exhibit rich diversity, with over 2000 strains recorded in GenBank as of April 02, 2026 (https://www.ncbi.nlm.nih.gov/labs/virus/vssi/#/). However, most of these phages have not been thoroughly characterized, leading to a lack of in-depth understanding of *S. aureus* phages. For example, the terminal structures of linear phage genomes are essential for DNA replication initiation and virion packaging (Dasgupta et al., 2024; Stewart et al., 1998); however, alignment of P108-related *S. aureus* phage genomes reveals substantial heterogeneity at their ends. Notably, despite this sequence divergence, all these phages are classified within the family *Herelleviridae* and genus *Kayvirus*—taxonomic groups whose members typically conserve terminal repeat architectures. This discrepancy indicated the need for further studies to determine the precise genome ends of these phages. Phylogenetic analysis of the whole-genome sequence places P108 as the closest relative to *S. aureus* phage Baghdad, whereas proteome-based phylogeny robustly clusters P108 with *S. aureus* phage G15. This topological incongruence suggests a mosaic genome architecture: P108 likely inherited its core genomic backbone from a Baghdad-like ancestor, while its structural protein module—central to proteomic phylogeny—was acquired via horizontal gene transfer or homologous recombination from a G15-related lineage (Nishimura et al., 2017a). Additionally, over half of the proteins encoded by the P108 genome remain functionally uncharacterized, constituting phage "dark matter" that may play a critical role in phage-host interactions. Multiple studies have identified a diverse array of phage-encoded proteins that play roles in bacterial defense mechanisms (Li et al., 2024a; Yirmiya et al., 2024; Yuan et al., 2025). Elucidating the functions of these uncharacterized proteins in phage P108 and related phages will likely uncover novel molecular mechanisms underlying phage-host interactions.

Furthermore, MRSA exhibits not only high pathogenicity and antibiotic resistance, but also a strong capacity for biofilm formation, posing significant challenges in clinical settings (Alexander et al., 2023; Collaborators, 2022; Miller and Arias, 2024). Over 60% of all infections are associated with biofilm formation, including those caused by *S. aureus* (James et al., 2008). Therefore, study of biofilm model is crucial when evaluating novel antibacterial phages. Previous studies have demonstrated that phages possess considerable potential in reducing or eliminating biofilms. For instance, phage K has been shown to significantly decrease the biofilm biomass of clinical *S. aureus* isolates upon application (Alves et al., 2014; Kelly et al., 2012). Our results indicated that phage P108 exerts a potent *in vitro* antibacterial effect comparable to that of vancomycin, and it can eliminate >80% of preformed biofilm, exhibiting a superior overall efficacy compared to vancomycin. The possible reason behind this might be that phages can more easily penetrate the dense biofilm matrix, are effective against dormant bacteria, and have the ability to actively multiply and spread (Cheng et al., 2025; Liu et al., 2024b).

Despite these promising findings, this study has certain limitations. The antibacterial activity was assessed solely *in vitro*, and its therapeutic efficacy *in vivo* remains to be validated. Moreover, the *in vivo* safety profile of phage P108 remains to be rigorously established. Future research should focus on *in vivo* models to evaluate the safety and effectiveness of P108, explore its synergistic effects with antibiotics, and engineer phages for enhanced bactericidal ability and host spectrum to optimize phage therapy applications (Ko et al., 2025; Olawade et al., 2024; Strathdee et al., 2023).

## 5 Conclusion

The high pathogenicity of *S. aureus* is not only attributed to its multidrug resistance, but also closely associated with its ability to form biofilms, evade host immune clearance, and cause persistent infections. To address this clinical challenge, the MRSA-targeting phage P108 was isolated and systematically characterized in terms of its morphology, thermal and pH stability, one-step growth curve, and 140,807 bp linear dsDNA genome. P108 was confirmed to be a stable, safe, and highly efficient lytic phage, classified as a member of the *Kayvirus* genus within the *Herelleviridae* family. Phage P108 exhibits dual therapeutic potential against MRSA infections: it effectively lysed 84.8% of clinical MRSA isolates and demonstrated significantly greater biofilm eradication capacity compared to vancomycin. The identification of P108 provides a promising strategy to combat chronic and refractory MRSA biofilm-associated infections and lays a solid foundation for the future development of phage-based therapies targeting highly pathogenic and drug-resistant bacteria.

## Data availability

The complete genome sequence and annotations of phage P108 have been deposited in GenBank under the accession number NC_025426.1 (identical to KM216423). Other data will be made available on request.

## CRediT authorship contribution statement

**Xuemei Wei:** Resources, Methodology, Writing – original draft, Software, Investigation, Data curation, Conceptualization. **Ruiyang Zhang:** Visualization, Methodology. **Jianglin Liao:** Data curation, Visualization. **He Liu:** Supervision, Resources. **Zhen Hu:** Validation, Supervision, Software. **Weilong Shang:** Resources, Project administration. **Jing Wang:** Funding acquisition, Conceptualization. **Ming Li:** Validation, Supervision, Resources. **Xiancai Rao:** Conceptualization, Supervision, Resources, Writing – review & editing. **Shuguang Lu:** Funding acquisition, Validation, Supervision, Resources, Project administration, Writing – review & editing.

## Declaration of competing interest

The authors declare that they have no known competing financial interests or personal relationships that could have appeared to influence the work reported in this paper.

## Appendix Supplementary materials

Image, application 1

Image, application 2

Image, application 3
