# Supplementary material for: Characterization and antibacterial efficacy of the broad-host-range phage P108 against biofilm-forming and methicillin-resistant Staphylococcus aureus
Source: Virus Res. 2026 May 12;368:199745. doi: 10.1016/j.virusres.2026.199745 (PMC13200134; doi:10.1016/j.virusres.2026.199745)
Supplement: Supplementary file 1 [file mmc1.pdf]

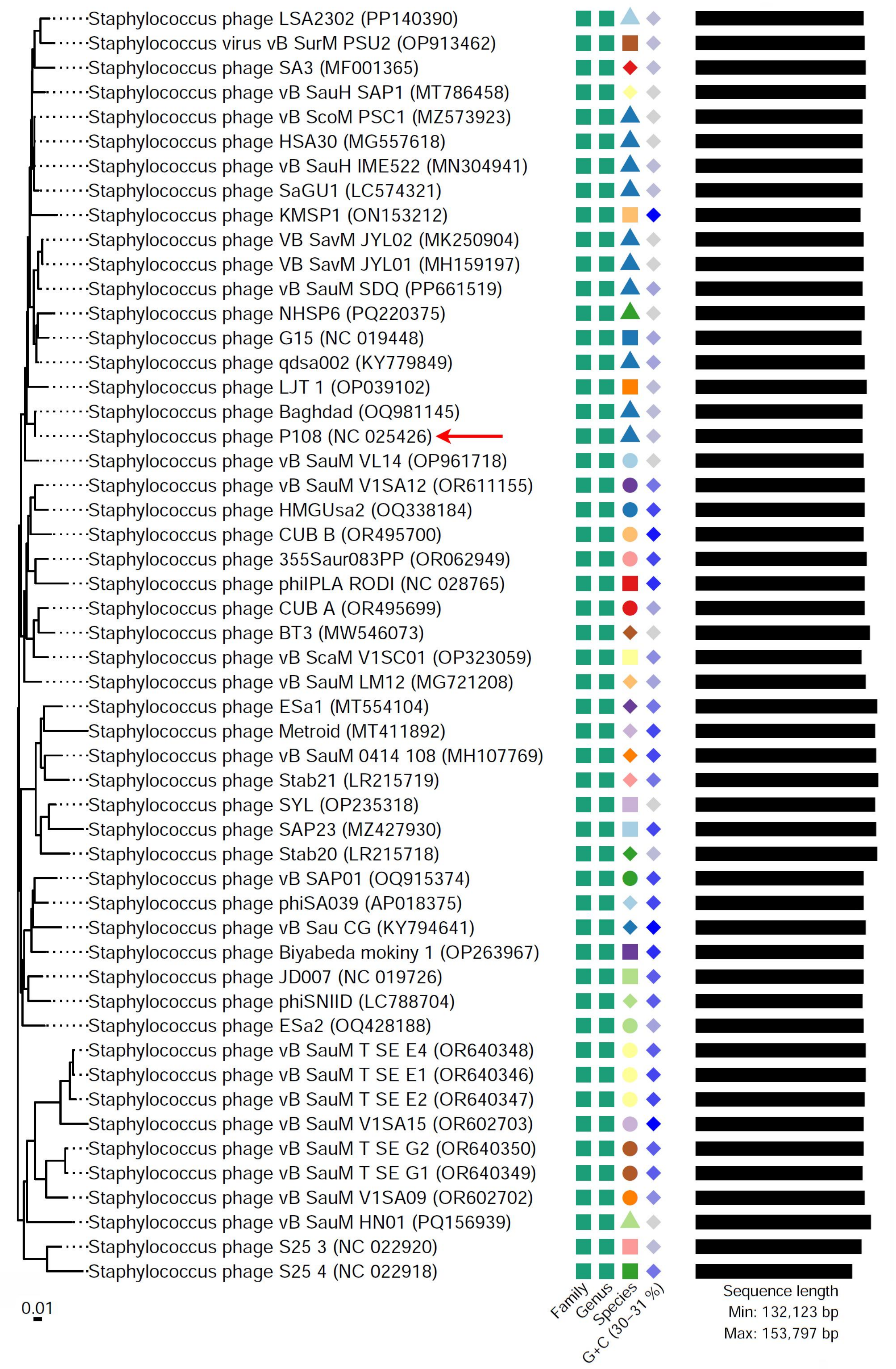

Fig. S1. Phylogenetic tree based on phage whole genome sequences. The red arrow indicates the position of phage P108. Scale bar: relative genetic distance (0.01).
