# Supplementary material for: Characterization and antibacterial efficacy of the broad-host-range phage P108 against biofilm-forming and methicillin-resistant Staphylococcus aureus
Source: Virus Res. 2026 May 12;368:199745. doi: 10.1016/j.virusres.2026.199745 (PMC13200134; doi:10.1016/j.virusres.2026.199745)
Supplement: Supplementary file 2 [file mmc2.pdf]

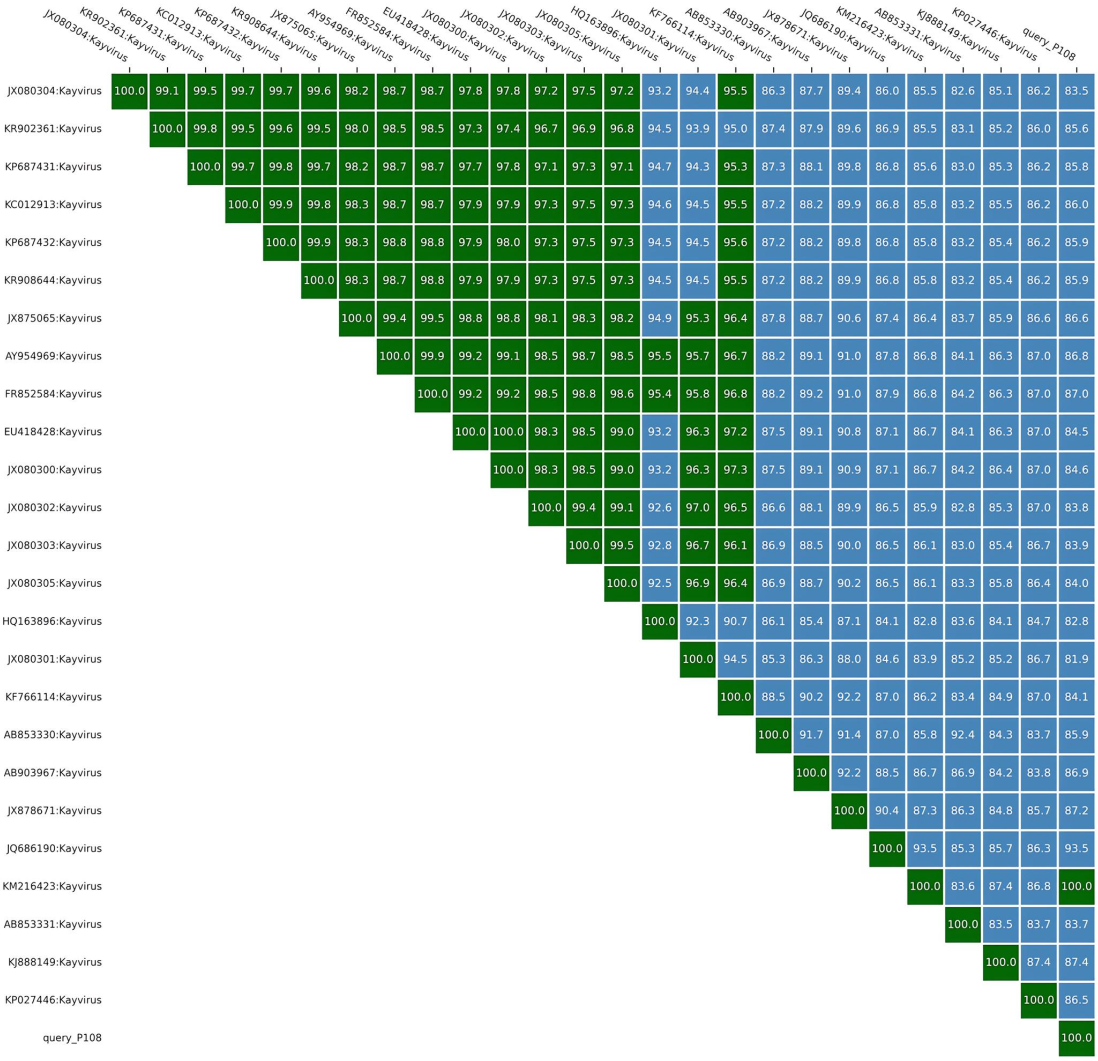

Fig. S2. Taxonomic analysis of phage P108 using taxMyPhage (https://ptax.ku.dk/). The numbers in the color blocks represent the average nucleotide identity (ANI) percentage. The GenBank accession number of phage P108 is NC_025426.1 (identical to KM216423).
